# Aberrant TMPRSS6‐Protease Regulation of Disease Mutant HCN4–KCNE1 Channel Complex Depends on the KCNE1‐G38S Polymorphism

**DOI:** 10.1002/ardp.70303

**Published:** 2026-07-09

**Authors:** David Linhoff, Minay Mertens, Ricarda Zimmermann, Stefan Peischard, Christoph Brenker, Thomas Budde, Sven Meuth, Christos Gatsogiannis, Annika Lüttjohann, Liudmila Sosulina, Jürgen Hescheler, Nathalie Strutz‐Seebohm, Guiscard Seebohm

**Affiliations:** ^1^ GRK 2515, Chemical Biology of Ion Channels (Chembion) Universität Münster Münster Germany; ^2^ Department of Cardiovascular Medicine Institute for Genetics of Heart Diseases (IfGH), University Hospital Münster Münster Germany; ^3^ Centre of Reproductive Medicine and Andrology, University Hospital Münster University of Münster Münster Germany; ^4^ Institut für Physiologie I Münster Germany; ^5^ Klinik für Neurologie mit Institut für Translationale Neurologie, ICB Münster Germany; ^6^ Institute for Medical Physics and Biophysics and Center for Soft Nanoscience Universität Münster Münster Germany; ^7^ Leibniz Institute for Neurobiology Magdeburg Germany; ^8^ Dr. Neher's Biophysics Laboratory for Innovative Drug Discovery, State Key Laboratory of Mechanism and Quality of Chinese Medicine, Faculty of Chinese Medicine Macau University of Science and Technology Macau SAR China

**Keywords:** environment, genetic, ion channel, modifier, proteolysis

## Abstract

The sinoatrial node pacemaker channel HCN4 plays a central role in cardiac automaticity, and disease‐associated variants can predispose to atrial arrhythmias. Here, we investigated the functional interplay between the HCN4 variant P883R and the potassium channel β‐subunit KCNE1, focusing on the common atrial fibrillation–associated KCNE1 variant G38S and its regulation by the iron‐induced serine protease TMPRSS6. Electrophysiological analyses revealed that HCN4‐P883R decreases net HCN4 currents *I*_f_, consistent with impaired automaticity. Co‐expression of KCNE1, either wild‐type or polymorphic KCNE1‐G38S, restored functional properties of the mutant channel, indicating that KCNE1 is a key modulator of HCN4 activity. Importantly, TMPRSS6‐mediated proteolytic processing of KCNE1 reduced HCN4 currents, whereas HCN4 expressed alone was insensitive to TMPRSS6, identifying KCNE1 as the direct regulatory target. Notably, KCNE1‐G38S altered the HCN4–KCNE1 complex to TMPRSS6‐dependent downregulation, resulting in a reduced suppression of HCN4‐P883R–mediated currents compared with wild‐type KCNE1. Mechanistically, differential TMPRSS6 cleavage depended on the membrane positioning of the KCNE1‐^32^RRSPRSS^38^ motif. These findings reveal a protease‐dependent buffering mechanism that counteracts HCN4 loss‐of‐function and establish TMPRSS6 as a molecular switch controlling pacemaker activity in a KCNE1 genotype–dependent manner. This dynamic regulatory framework may contribute to the phenotypic variability of sinoatrial node dysfunction and atrial fibrillation.

## Graphical abstract

TMPRSS6‐mediated cleavage of the HCN4–KCNE1 channel complex may modulate disease phenotype in a KCNE1 genotype‐dependent manner.

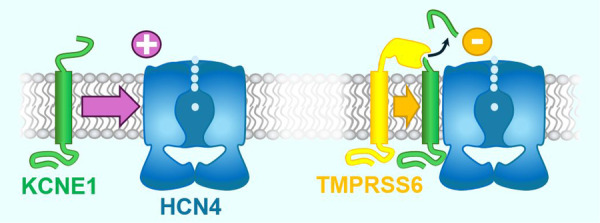

## 1 Introduction

The sinoatrial node (SAN) is the primary pacemaker of the heart and ensures highly controlled rhythmic excitation and autonomic modulation through the tightly regulated interplay of key ion channels [1]. Functional disturbances of SAN function result in sick sinus syndrome (SSS), a clinically heterogeneous disorder characterized by sinus bradycardia, unstable sinus rhythms, chronotropic incompetence, and frequently alternating episodes of atrial tachyarrhythmias like atrial fibrillation (AF) [2, 3, 4, 5, 6]. Beyond symptomatic bradyarrhythmias, SAN dysfunctions are increasingly recognized as part of a broader atrial and ventricular disease spectrum, linking primary pacemaker abnormalities to atrial cardiomyopathy, AF susceptibility, and structural heart disease as well [7]. Genome‐wide association studies and candidate gene analyses have identified numerous loci associated with AF and/or SAN dysfunction, many of which encode ion channels or proteins involved in cardiac excitability and conduction [8, 9]. Rather than acting as monogenic causes, many variants function as disease modifiers that subtly alter electrophysiological properties and lower the threshold for arrhythmia initiation and maintenance [10, 11]. Among these, hyperpolarization‐activated cyclic nucleotide‐gated (HCN) channels, particularly HCN4, have emerged as central regulators of cardiac automaticity and arrhythmogenesis. HCN4 is the predominant HCN isoform in the human SAN and generates the pacemaker current I_f_, a mixed Na^+^/K^+^ inward current activated upon hyperpolarization and modulated by cyclic adenosine monophosphate (cAMP) [12]. *I*_f_ is the key player controlling diastolic depolarization to determine the heart rate and autonomic control of pacemaker activity [13, 14]. Loss‐of‐function mutations in HCN4 were initially linked to inherited sinus bradycardia and SSS [15, 16], but subsequent studies expanded the phenotypic spectrum to include AF, ventricular arrhythmias, and cardiomyopathies such as left ventricular noncompaction [6, 7]. Mechanistically, HCN4 dysfunction affects not only impulse initiation in the SAN but also ectopic pacemaker activity in atrial tissue. Both experimental and clinical data suggest that alterations in HCN4 gating, current density, or cAMP sensitivity can promote atrial ectopy and facilitate AF initiation [17, 18, 19]. Consistently, variants in HCN4 and interacting proteins were identified by GWAS and are associated with increased AF risk, further underscoring the relevance of this channel in atrial electrophysiology [8, 18, 19, 20]. Beyond arrhythmia initiation, sustained atrial tachyarrhythmias can lead to tachycardia‐induced cardiomyopathy (TIC), a potentially reversible form of structural heart disease characterized by ventricular dysfunction secondary to chronic high heart rates [7, 21]. Although TIC is clinically well recognized, the molecular determinants that predispose certain patients with AF to develop ventricular remodeling remain poorly understood. Very recently, structural‐electrical remodeling was linked to Coxsackie virus B3 (CVB3) myocarditis [22]. Reduced HCN4 function by cardiotropic CVB3 was reported in murine hearts and human iPSC‐cardiac pacemaker tissue models linking HCN4 function to environmental cues [23]. Recent genetic studies have implicated HCN4 variants in the susceptibility to TIC, suggesting that altered pacemaker currents may contribute not only to electrical instability but also to maladaptive structural remodeling [7]. In this context, the C‐terminal HCN4 variant P883R has attracted particular attention. Located distal to the cyclic nucleotide‐binding domain (CNBD), P883R affects a region involved in channel gating, subunit interaction, and autoinhibition [1, 24]. Functional characterization demonstrated that HCN4‐P883R shifts the voltage dependence of activation toward more depolarized potentials, accelerates deactivation, and, when co‐expressed with wild‐type (WT) subunits, increases current density, thereby increasing the net *I*_f_ current [7]. Clinically, this variant has been associated with AF, SSS, and structural heart disease, supporting its role as a genetic modifier rather than a sole causative mutation. Importantly, carriers of HCN4‐P883R were found to share a common polymorphic variant of KCNE1 [7], encoding the β‐subunit of the slow delayed rectifier potassium current *I*_Ks_. KCNE1 is best known for its crucial role in cardiac repolarization by modulating KCNQ1 channel kinetics, surface expression, and voltage dependence [25, 26]. Variants in KCNE1 have been associated with congenital and acquired long QT syndrome, AF, and altered atrial refractoriness [27]. The common polymorphism KCNE1‐G38S has been repeatedly linked to AF susceptibility (SNP: rs1805127; T > C transition; G38S substitution), and it was discussed to reduce *I*_Ks_, thereby prolonging action potential duration and diminishing repolarization reserve [27, 28, 29, 30]. Herlyn et al. report on gene frequencies of these common polymorphisms in central Europeans (0.383), Han Chinese/Beijing (0.400), Japanese, Tokyo (0.295), and Yoruba/Nigeria (0.3), suggesting that about 1/3 of people carry the KCNE1‐G38S variant [31]. Interestingly, the Kora study found no association of variant KCNE1‐G38S to QT interval in Central European Caucasians [32], suggesting that KCNE1 may have additional relevant interacting protein partners that are relevant for AF. Recently, we showed in a preprint that KCNE1 can increase HCN4 current [33]. From a pathophysiological perspective, the combination of increased inward depolarizing current (*I*_f_) and reduced outward repolarizing current (*I*_Ks_) represents a pro‐arrhythmic substrate that may facilitate early and delayed afterdepolarizations, enhance ectopic firing, and stabilize reentrant circuits. Computational modeling and experimental studies indicate that KCNE1‐mediated *I*_Ks_ reduction alone may be less sufficient in inducing arrhythmia but becomes highly arrhythmogenic in the presence of additional depolarizing influences or calcium‐handling disturbances, and thus, concomitant functionally relevant HCN4 effects may provide an additional trigger that converts a vulnerable substrate into manifest arrhythmia [34]. Beyond genetic variation in pore‐forming and auxiliary ion channel subunits, posttranslational regulation has emerged as a critical yet underappreciated determinant of cardiac electrophysiology. Proteolytic processing of ion channel complexes represents a powerful mechanism to dynamically fine‐tune channel availability, gating behavior, and subcellular localization, thereby shaping cardiac excitability under physiological and pathological conditions [35]. Transmembrane protease serine 6 (TMPRSS6), a type II transmembrane serine protease best known for its role in systemic iron homeostasis, has recently been identified as a novel regulator of cardiac ion channels. Notably, TMPRSS6 was shown in a preprint to proteolytically cleave the KCNE1 accessory subunit, resulting in altered *I*_Ks_ channel and *I*_f_ channel function and surface expression [33]. Proteolytic truncation of KCNE1 modifies its modulatory control over Kv7.1 and HCN4, leading to significant changes in repolarization dynamics via *I*_Ks_ and bradycardia via *I*_f_ reduction [33]. These findings establish TMPRSS6 as an additional layer of functional regulation of *I*_Ks_ and *I*_f_ beyond genetic variations. Importantly, the efficiency and functional consequences of TMPRSS6‐mediated KCNE1 cleavage appear to depend on the molecular context of KCNE1, suggesting that common KCNE1 variants may differentially respond to proteolytic processing. Given that the KCNE1‐G38S variant is associated with AF and reduced repolarization reserve [27, 28, 30], altered susceptibility to TMPRSS6‐dependent cleavage could further amplify its arrhythmogenic potential. Such a mechanism would provide a compelling explanation for interindividual variability in electrophysiological phenotypes among carriers of identical KCNE1 genotypes. Taken together, these observations suggest a complex, multilevel regulatory network in which genetic variation (HCN4‐P883R and KCNE1‐G38S) and posttranslational modification (TMPRSS6‐mediated KCNE1 cleavage) may converge to shape cardiac pacemaker activity and repolarization. However, it remains unknown how TMPRSS6‐dependent processing of KCNE1 affects HCN4‐mediated pacemaker currents, and whether disease‐associated KCNE1 variants modify this interaction. In particular, the impact of KCNE1 cleavage on HCN4–KCNE1 functional coupling, and its dependence on KCNE1 genotypic background, has not been systematically investigated. Therefore, the present study aims to elucidate the molecular and electrophysiological interplay between HCN4 and KCNE1 (WT vs. G38S) under conditions of TMPRSS6‐mediated proteolytic regulation. By integrating genetic, posttranslational, and functional perspectives, this work seeks to define a novel mechanism linking pacemaker dysfunction, atrial arrhythmogenesis, and susceptibility to SS and AF. A deeper understanding of these converging regulatory layers may ultimately enable more precise stratification of arrhythmic risk and identify new molecular targets for individualized therapeutic intervention.

## 2 Results and Discussion

### 2.1 Both Predominant KCNE1 Variants, KCNE1‐WT and ‐G38S Can Correct HCN4‐P883R Misfunction Associated Functional Defect

HCN4‐P883R was reported to cause altered function [7]. However, the molecular basis of these functional changes in HCN4‐P883R remains unclear. Typical reasons for loss of function in disease‐associated ion channel variants include protein functional and trafficking defects that cause reduced plasma membrane expression, leading to reduced channel function at the plasma membrane [19]. Recently, we reported that the accessory ion channel subunit KCNE1 can boost membrane expression and function of HCN4‐WT channels. Therefore, the effect of coexpression of the two most common KCNE1 variants and HCN4‐WT or HCN4‐P884R channels variant was analyzed by two‐electrode voltage‐clamp (TEVC).

**Figure 1:**
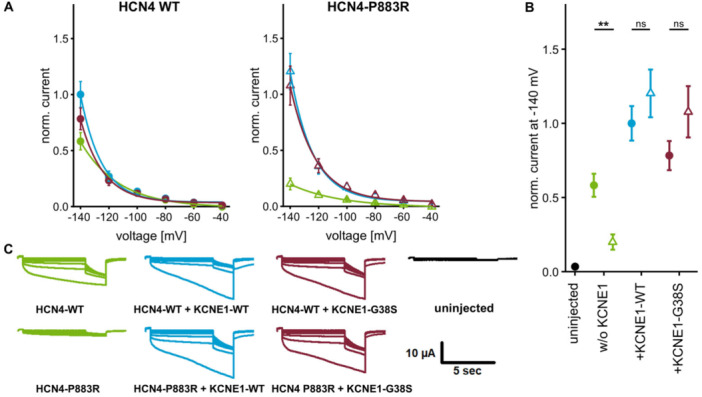
HCN4–KCNE1 variants in TEVC measurements in *Xenopus laevis* oocytes. Current–voltage (*I*–*V*) relationships (A) and normalized current amplitudes at −140 mV. Currents were elicited by 5‐s hyperpolarising pulses ranging from −40 to −140 mV in 20 mV decrements and followed by −60 mV tail pulses. (B) for HCN4‐WT, HCN4‐P883R or co‐expressed with KCNE1‐WT or KCNE1‐G38S. Currents were normalized to the HCN4‐WT + KCNE1‐WT, where filled circles represent HCN4‐WT and the open symbols indicate KCN4‐P883R. (C) Representative TEVC recordings. Co‐expression with KCNE1 enhanced currents versus single HCN4 expression and nullified the difference between HCN4‐WT and HCN4‐P883R. Data were presented as mean ± SEM (*n* = 10–13 per group), statistical differences (*p* ≤ 0.01) were indicated by **.

The modulatory effects of the KCNE1 WT (KCNE1‐WT) (present in about 2/3 of populations) and the KCNE1‐G38S (present in about 1/3 of populations) subunits on both HCN4‐WT and the HCN4‐P883R mutant channels were investigated using TEVC recordings in *Xenopus laevis* oocytes (Figure 1). Expression of the HCN4‐WT alone resulted in a typical hyperpolarisation‐activated current profile. At −140 mV, the average current of HCN4‐WT was −12.25 ± 1.64 µA, whereas HCN4‐P883R showed only −4.19 ± 1.08 µA, which is ≈34% of the HCN4‐WT (*p* < 0.01). Coexpression with KCNE1‐WT tended to increase current amplitudes of HCN4‐WT by ≈72% (−21.03 ± 2.44 µA, *p* = 0.07) and rescued the HCN4‐P883R current to −25.29 ± 3.38 µA, a ≈6‐fold augmentation relative to HCN4‐P883R alone (*p* ≤ 0.001). The mutant KCNE1‐G38S also enhanced currents, albeit to a slightly lower extent (HCN4‐WT + KCNE1‐G38S: −16.46 ± 2.05 µA, *p* > 0.05; HCN4‐P883R + KCNE1‐G38S: −22.66 ± 3.64 µA, *p* < 0.01). Importantly, the currents of HCN4‐WT + KCNE1 and HCN4‐P883R + KCNE1 exert no significant differences, uncovering a recovery of function in disease mutant HCN4‐P883R by KCNE1 (*p* > 0.05). The KCNE1‐mediated rescue of HCN4‐P883R showed no significant difference between KCNE1‐WT and the common polymorphic variant KCNE1‐G38S (*p* > 0.05). Thus, loss‐of‐function in a disease‐associated HCN4 channel variant can be corrected by the KCNE1 subunit up to a level similar to that of HCN4‐WT co‐expressed with KCNE1, and therefore, KCNE1 can be regarded as a modulator of disease‐associated functional phenotype.

### 2.2 TMPRSS6 Affects HCN4 Variant Current Amplitudes Depending on KCNE1 Genotype

**Figure 2:**
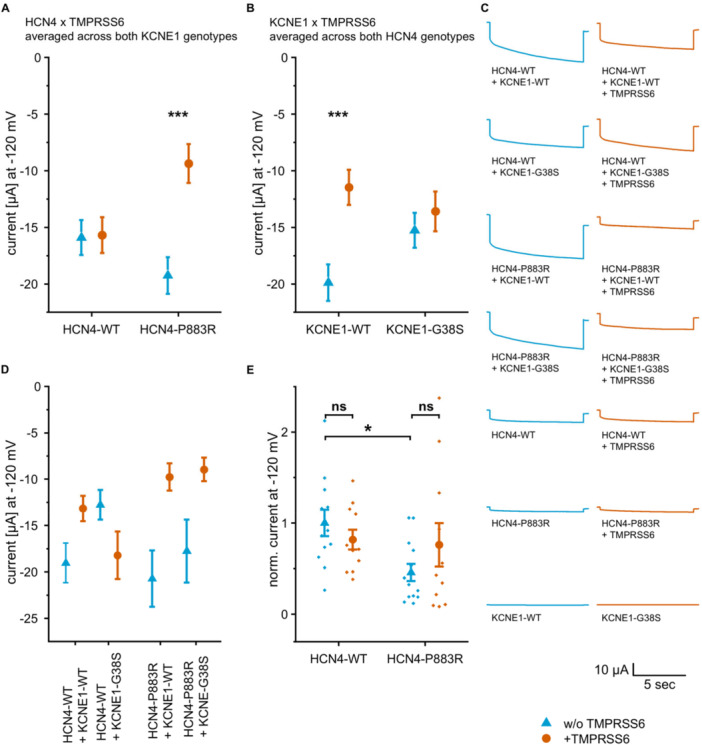
TMPRSS6 effect on HCN4/KCNE1 current amplitudes at −120 mV. Combinations of HCN4 (WT and P883R) × KCNE1 (WT and G38S) × TMPRSS6 (−/+) co‐expression in *Xenopus laevis* oocytes. Interaction plots of TMPRSS6 effect on HCN4‐WT versus ‐P883R currents at −120 mV averaged over KCNE1 genotype (A) and TMPRSS6 effect on KCNE1‐WT versus ‐G38S currents at −120 mV averaged over HCN4 genotype (B). Blue triangles represent groups without TMPRSS6, and orange circles indicate co‐expression with TMPRSS6. (C) Example measurements of the tested conditions. (D) Overview of the current amplitudes at −120 mV of the tested groups. (E) Current amplitudes at −120 mV of HCN4 (WT and P883R) ± TMPRSS6 normalized to HCN4‐WT show no significant effect of TMPRSS6 on the channel alone. Data were presented as mean ± SEM (*n* = 7–13 per condition), statistical differences are indicated as follows: *p < 0.05, ***p < 0.001.

It has been shown in a preprint that TMPRSS6 reduces HCN4/KCNE1 current amplitudes via cleavage of KCNE1 [33]. We analyzed current amplitudes at −120 mV in a 2 × 2 × 2 experimental design for testing combinations of HCN4 (WT vs. P883R), KCNE1 (WT vs. G38S) × TMPRSS6 (+/−) using TEVC in *X. laevis* oocytes (Figure 2). The impact of TMPRSS6 on current amplitudes differed depending on the HCN4 channel genotype (three‐way ANOVA, interaction of HCN4 × TMPRSS6, *p* < 0.01). The HCN4‐P883R mutant coexpressed with KCNE1 markedly increased TMPRSS6 sensitivity, reducing currents by 51% (−19.25 ± 1.62 µA to −9.38 ± 1.72 µA; *p* < 0.001). In contrast, the HCN4‐WT + KCNE1 × TMPRSS6 interaction showed a negligible effect under these conditions (−15.91 ± 1.55 µA to −15.70 ± 1.58 µA; *p* > 0.05).

Furthermore, we found different influences of TMPRSS6 depending on the KCNE1 genotype (three‐way ANOVA, interaction of KCNE1 × TMPRSS6, *p* < 0.01). In case of WT KCNE1, TMPRSS6 lowered the current by ≈42% (−19.89 ± 1.62 µA to −11.48 ± 1.55 µA; *p* < 0.001), while in case of KCNE1‐G38S, however, this TMPRSS6 effect was only around 11% (−15.27 ± 1.55 µA to −13.59 ± 1.75 µA; *p* = 0.47).

These observations suggest that TMPRSS6‐mediated proteolysis of KCNE1 impairs HCN4 currents in a manner dependent on the specific KCNE1 genotype.

The HCN4‐P883R mutant without KCNE1 exhibits significantly reduced current amplitudes at −120 mV compared to the HCN4‐WT (HCN4‐WT: −7.93 ± 1.15 µA; HCN4‐P883R: −3.63 ± 0.74 µA; *p* < 0.05). Under these conditions, TMPRSS6 co‐expression shows no significant current effect on the HCN4 channel alone. This demonstrates that the TMPRSS6 effect is mediated via cleavage of KCNE1 (Figure 2E).

### 2.3 Kv7.1‐KCNE1‐WT and Kv7.1‐G38S Show Similar TMPRSS6 Sensitivities

**Figure 3:**
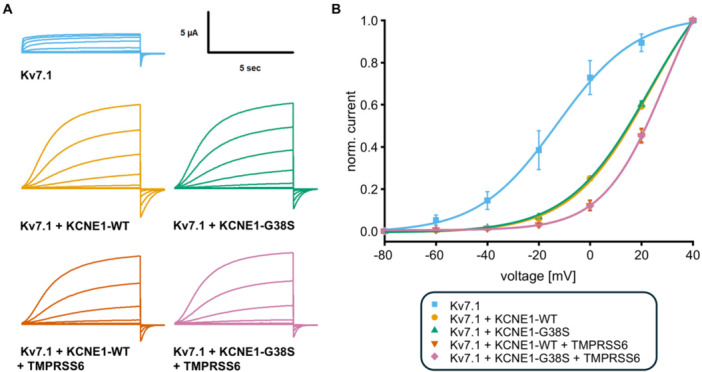
Influence of TMPRSS6 on voltage‐sensitivity of Kv7.1/KCNE1 currents. (A) Example traces for the different recording conditions. Currents were elicited by 7‐s depolarizing pulses ranging from −80 to +40 mV in 20 mV increments and followed by −120 mV tail pulses. (B) Current amplitudes were normalized to +40 mV and plotted against the applied voltage. TMPRSS6‐proteolytic domain (PD) shifts the activation curve of Kv7.1/KCNE1 to more depolarized potentials. As there was no relevant difference between the co‐expression of KCNE1‐WT and ‐G38S when TMPRSS6 was present or absent, the respective curves overlap (Kv7.1 alone: *n* = 3; all other groups: *n* = 8–12).

KCNE1 acts as an auxiliary ß‐subunit of Kv7.1 channels, thereby shifting kinetics and voltage dependence. We examined the impact of TMPRSS6 on Kv7.1/KCNE1 (WT vs. G38S) channel function in *X. laevis* oocytes using TEVC (Figure 3). Kv7.1 alone shows rapid voltage‐dependent activation with peak conductance at positive potentials. Co‐expression with KCNE1 resulted in classical right‐shifted voltage‐dependency with no difference between KCNE1‐WT or ‐G38S. In the groups of Kv7.1 and KCNE1 (WT or G38S) with TMPRSS6 proteolytic domain (PD), an additional right‐shift was observed. Kv7.1‐KCNE1‐WT and Kv7.1‐KCNE1‐G38S showed similar TMPRSS6‐PD sensitivities under these experimental conditions.

### 2.4 Proteolysis of KCNE1 Variants Depends on the KCNE1 Genotype and Presence of Physiologically Relevant Iron Concentrations

**Figure 4:**
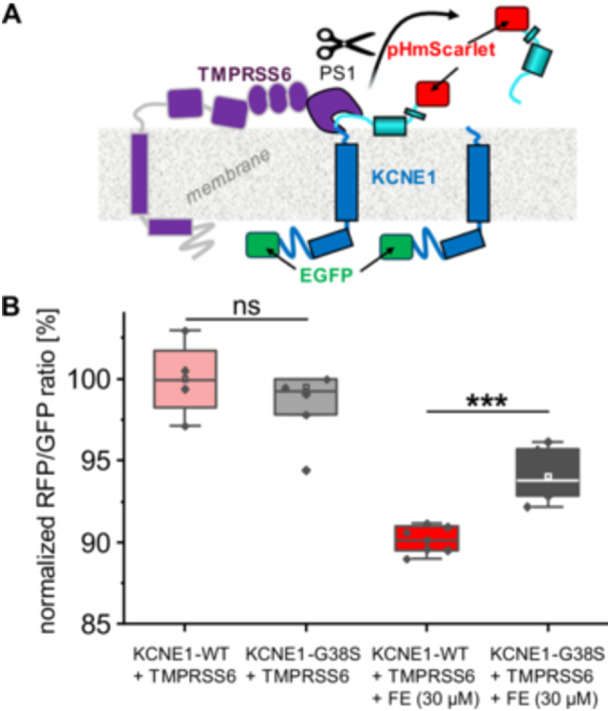
FACS assay for quantifying the proteolysis efficiency of KCNE1 by TMPRSS6 at physiological iron concentration. (A) Illustration of the FACS assay principle. HEK cells were transfected with TMPRSS6 and pHmScarlet‐KCNE1‐EGFP cDNA constructs. TMPRSS6 cleavage of N‐terminal red pHmScarlet from the KCNE1‐EGFP was analyzed by FACS to assess the relative cleavage efficiency of TMPRSS6 per cell (red‐to‐green fluorescence intensity ratio = RFP/GFP ratio). Since TMPRSS6 cleavage releases an extracellular fragment of KCNE1 that contains pHmScarlet, a lower RFP/GFP ratio indicates higher cleavage activity. (B) The RFP/GFP ratio per transfection approach was normalized to mean values of KCNE1‐WT + TMPRSS6. Addition of iron (FE) increased the TMPRSS6 cleavage efficiency of KCNE1‐WT more than that of KCNE1‐G38S. Statistical differences (*p* ≤ 0.001) are indicated by *** (*n* = 4–6 per condition).

Our TEVC recordings revealed that the functional impact of TMPRSS6 on HCN4/KCNE1 currents depends on the specific KCNE1 genotype (Figure 2). We next investigated the mechanistic basis of this difference using a direct TMPRSS6 cleavage assay (Figure 4). This FACS‐based assay revealed that TMPRSS6 cleaves the KCNE1‐WT more efficiently than the KCNE1‑G38S variant. The assay principle was described previously in a preprint [20]. Fluorescent proteins were attached to KCNE1, an EGFP (green) to the intracellular C‐terminal part, and a pHmScarlet (red) to the extracellular N‐terminal part. PHmScarlet fluorescence is pH sensitive, so that only KCNE1 expressed at the cell surface was analyzed. PHmScarlet‐KCNE1 in cellular vesicles was largely reduced due to pH decrease, increasing the focus of analyses to the plasma membrane [36]. As TMPRSS6 cleaves KCNE1 extracellularly, proteolytic processing results in the loss of the extracellular pHmScarlet tag. The intracellular EGFP is associated with the remaining KCNE1 construct, acting as an indicator for total KCNE1 expression. Therefore, the red‐to‐green fluorescence intensity ratio (RFP/GFP ratio) was inversely related to TMPRSS6 cleavage efficiency at the plasma membrane.

The RFP/GFP ratio per cell was calculated in the FACS results of the EGFP positive cell‐fraction, averaged per transfection approach, and normalized to KCNE1‐WT + TMPRSS6. Under basal conditions, this ratio between KCNE1‐WT and KCNE1‐G38S was similar. Incubation with 30 µM iron (FE) as a medium to high‐physiological concentration corresponding to the upper end of normal adult serum iron range (11–30 µM) enhanced TMPRSS6‐mediated cleavage of KCNE1 with polymorphism‐specific differences. The normalized RFP/GFP ratio of KCNE1‐WT + FE was decreased by 10% (1.00 ± 0.01–0.90 ± 0.00; *p* < 0.001), indicating a higher TMPRSS6 cleavage efficiency under iron exposure. The polymorphic KCNE1‐G38S + FE showed a smaller 6% decrease in the RFP/GFP ratio (1.00 ± 0.02–0.94 ± 0.01; *p* = 0.01). The two groups differed significantly when iron was added (*p* < 0.001), suggesting that TMPRSS6 cuts KCNE1‐WT more than KCNE1‐G38S.

### 2.5 TMPRSS6 Proteolysis of KCNE1 Depends on Precise Positioning of KCNE1 Outer Segment in Relation to the Outer Plasma Membrane Edge

**Figure 5:**
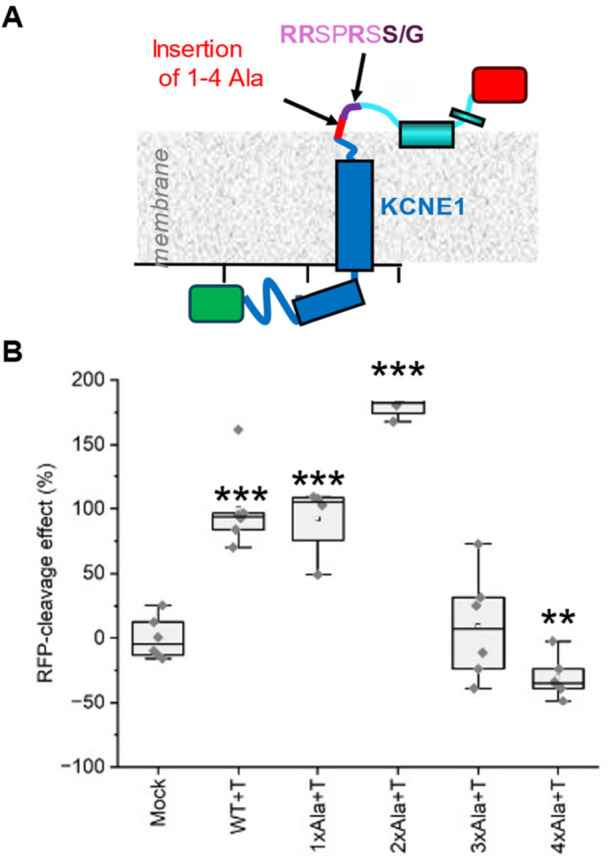
Importance of precise positioning of the recognition segment (RRSPRSS) within KCNE1 for its proteolysis by TMPRSS6. (A) Illustration of the FACS assay. 1–4 alanines (Ala, red) were inserted into pHmScarlet‐KCNE1‐EGFP constructs between the RRSPRSS sequence (magenta) and the outer membrane leaflet. (B) TMPRSS6‐cleavage was identified as an increase in the cell fraction showing low RFP intensity (RFP‐cleavage effect) in transfected HEK cells. TFP cleavage was normalized to mean WT + T and scaled to 100%. Statistical differences (*p* ≤ 0.01; *p* ≤ 0.001) are indicated by **; *** (*n* = 4–6 per condition).

Similar to the FACS assay used to evaluate proteolysis of KCNE1‐WT and ‐G38S, we analyzed how the exact membrane localization affects TMPRSS6‐dependent cleavage of KCNE1 (Figure 5). One to four alanines (Ala) were introduced into pHmScarlet‐KCNE1‐EGFP constructs immediately preceding the previously described TMPRSS6 (T) recognition sequence (^32^RSPRSG/S^38^; G/S represent polymorphic residue 38) (Figure 5). These KCNE1 constructs and TMPRSS6 were co‐transfected in HEK cells, whereas the mock control was transfected with an empty pcDNA3 vector. Cleavage by TMPRSS6 was detected as an increase in the cell population exhibiting reduced RFP fluorescence. Results were normalized to KCNE1‐WT + T. Notably, the cleavage effect in the 2 × Ala + T group exceeded that of the WT by 173.43 ± 8.26% (*p* ≤ 0.001), indicating that positioning of the cleavage site in WT and G38S may not be perfect for TMPRSS6 proteolysis. No cleavage effect was observed when three or more alanines were inserted.

### 2.6 In Silico Interactions of KCNE1‐WT and KCNE1‐G38S Reveal Altered Interactions With TMPRSS6

**Figure 6:**
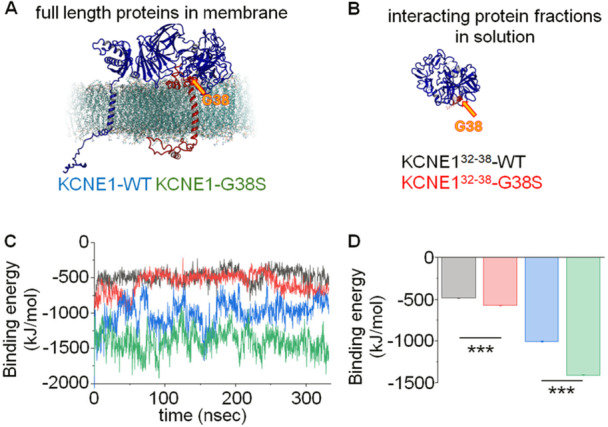
Molecular dynamics simulations uncover dependence of binding energies on KCNE1 residue 38. All‐atom mobile molecular dynamics simulations were run for 331 ns and conducted on (A) full‐length TMPRSS6/KCNE1‐WT and KCNE1‐G38S in membrane environment and (B) on partial TMPRSS6 with bound KCNE1^32–38^ peptides in 0.9% NaCl. The simulation conditions are depicted above, whereas the position of KCNE1 residue G38 is indicated in orange. (C) The binding energies of KCNE1 to TMPRSS6 were calculated over simulation times (lower left), and (D) the mean ± SEM was calculated (lower right). Student's *t*‐test suggests highly significant differences in binding energies in silico.

To combine the experimental data with structural modeling analyses of the peptide/protease complex, we used a recently constructed TMPRSS6‐KCNE1 protein complex model [33]. The position of the KCNE1 key region ^32^RRSPRSG/S^38^ with respect to the substrate cleft of the TMPRSS6 protease was determined by analogous positioning to the peptidomimetic benzothiazole that was previously co‐crystallized with the homologous peptidic matriptase‐1 (TMPRSS2) (PDB ID *6N4T*). Molecular dynamics (MD) simulations were then performed on TMPRSS6‐KCNE1‐WT and TMPRSS6‐KCNE1‐G38S in a membrane in parallel, and simulation trajectories were subsequently analyzed (Figure 6). The simulations revealed that the KCNE1^32–38^ region and TMPRSS6 form a stable complex that is anchored to the outer membrane edge (outer membrane leaflet). The KCNE1^41–67^ form an α‐helix stably inserted into the membrane, as in silico and experimentally shown previously [37, 38]. The N‐terminal transmembrane α‐helix TMPRSS6^46–70^ anchors the TMPRSS6 complex to the membrane [33]. During the production runs, the KCNE1 32–38 adopted a stable conformation within the catalytic peptidase S1 domain without disrupting the active side of the domain. Analyses of the interaction endurance between KCNE1 and TMPRRS6 show that the interactions cluster H608, D659, D747, V772, S773, W774, G775, and G777 around the proteolytic residue S753 are stable during simulations. KCNE1 R36 forms very stable H‐bonds and/or ionic interactions with TRPMSS6 D747 and G777, which may allow for nucleophilic attack and subsequent proteolytic cleavage in vitro. However, direct comparison of simulations of the variants seemed to exhibit complex dynamic differences. To quantify interaction energies in this complex system, the binding energies of KCNE1 to TMPRSS6 in a membranous environment were analyzed. Clear differences in interaction energies were detected (note: lower values indicate increased binding energies). KCNE1‐WT binding energy: −1007.2 ± 5.2 kJ/mol versus KCNE1‐G38S: −1407.3 ± 4.6 kJ/mol, suggesting that serine at KCNE1 position 38 favors stable interactions with TMPRSS6 and might introduce an energetic barrier into the proteolytic cycle, reducing the proteolytic rate. As these differences in interactions could be due to less interaction of peptide KCNE1^32–38^ with the TMPRSS6 PD and/or due to different positioning of the KCNE1^32–38^ regions relative to the outer membrane/TMPRSS6, simulations of the isolated TMPRSS6 PD and the isolated KCNE1^32–38^ were conducted. The residues TMPRSS6^1–450^ were deleted, and the simulations were run in physiological 0.9% NaCl without membranes. Interestingly, the peptide KCNE1‐WT showed less affinity in silico (−482.9 ± 2.8 kJ/mol) compared to the KCNE1‐G38S peptide (−571.6 ± 3.8 kJ/mol) in this reduced model system. This might indicate that KCNE1‐G38S introduces an energetic barrier into the catalytic cycle due to increased interaction of the intermediate state KCNE1‐G38S‐TMPRSS6, which may lead to reduced proteolytic rate.

## 3 Discussion and Conclusion

In the present study, we investigated the functional interplay between the SAN‐associated HCN4 variant P883R and the potassium channel β‐subunit KCNE1, with particular emphasis on the differential effects of the common AF‐associated KCNE1‐G38S variant [27, 28, 30] and its regulation by the serine protease TMPRSS6 as shown in a preprint [33]. Our findings uncover a previously unrecognized mechanism by which KCNE1 genotype and proteolytic processing jointly modulate HCN4 function, thereby shaping cardiac automaticity and arrhythmia susceptibility. Consistent with previous reports, HCN4‐P883R modulates net *I*_f_ [7]. Importantly, however, our data indicate that the functional impact of HCN4‐P883R is not fixed, but rather subject to modulation by the molecular environment in which the channel operates. This observation is critical, as it suggests that disease‐associated HCN4 variants may not act as constitutive loss‐of‐function mutations, but instead participate in a dynamic regulatory network that can either amplify or restrain pacemaker activity depending on contextual factors.

A central novel finding of this study is that disease mutant HCN4‐P883R can be recovered by common forms of KCNE1 (WT and G38S) (Figure 1), suggesting that KCNE1 expression is a key factor of *I*_f_ in both WT and disease mutant settings. We have shown before in a preprint that TMPRSS6‐mediated proteolytic processing of KCNE1 reduces the stimulation of HCN4 in heterologous expression and in human iPSC‐derived pacemaker cardiomyocytes [33]. Our current data show a similar reduction of HCN4/KCNE1 by TMPRSS6, whereas the HCN4 channel expressed alone is not sensitive to TMPRSS6, supporting the hypothesis that KCNE1 is the direct target of TMPRSS6 regulation (Figure 2E). However, KCNE1‐G38S, under TMPRSS6‐mediated proteolytic processing, exerts a weakened effect on HCN4‐P883R compared to HCN4‐WT. Thus, in contrast to KCNE1 WT, the KCNE1‐G38S variant desensitizes the HCN4–KCNE1 system to TMPRSS6‐dependent downregulation, resulting in relative increased *I*_f_ of this disease‐associated mutant channel. TMPRSS6 emerges from our study as a critical regulatory node that determines the net electrophysiological outcome of functional HCN4–KCNE1 interactions. By proteolytically processing KCNE1, TMPRSS6 indirectly controls HCN4 abundance and/or stability in a KCNE1 genotype‐dependent manner. In the presence of KCNE1‐G38S, TMPRSS6‐mediated cleavage results in increased mutant *I*_f_, thereby attenuating and counteracting the intrinsic loss‐of‐function properties of HCN4‐P883R. This mechanism introduces the concept of protease‐dependent regulation of pacemaker function. Rather than uniformly exacerbating arrhythmia susceptibility, TMPRSS6 activity may restrain excessive automaticity in certain genetic contexts. Such an effect could explain the marked interindividual variability observed among carriers of HCN4 variants, including differences in heart rate, AF burden, and progression to structural heart disease.

Therefore, KCNE1‐G38S does not merely act as a passive modifier of repolarization through I_Ks_, but actively introduces an additional inhibitory component within the pacemaker apparatus. This observation adds a new dimension to the functional role of KCNE1. While KCNE1‐G38S has traditionally been viewed as a loss‐of‐function variant that reduces *I*_Ks_ and thereby promotes arrhythmogenesis by impairing repolarization reserve, our data demonstrate that—in the presence of TMPRSS6—it can simultaneously suppress HCN4‐dependent automaticity. This duality suggests that KCNE1‐G38S may exert both pro‐arrhythmic and anti‐automatic effects, depending on the balance between depolarizing and repolarizing currents and on protease activity. The Kora study failed to associate variant KCNE1‐G38S with the QT interval in Central European Caucasians [32]. Consistently, we detect no altered TMPRSS6 sensitivity in Kv7.1/KCNE1‐G38S compared to Kv7.1/KCNE1‐WT (Figure 3E). This finding suggests that KCNE1 may have additional relevant interacting protein partners than Kv7.1 that are relevant for AF. This partner could potentially be the HCN4 in a functional modulated protein network that is sensitive to genetic variation. However, more research is required to elaborate on this hypothesis. Summarizing, TMPRSS6 acts as a molecular switch between enhanced and restrained automaticity.

Our data suggest a revised model of HCN4‐linked arrhythmogenesis based on dynamic balance rather than unidirectional gain‐of‐function in HCN4 via KCNE1. The key to this revised pathophysiological model of atrial arrhythmia susceptibility arises from a dynamic balance between two opposing forces: Pro‐automatic influences in mutants, such as HCN4‐P883R‐mediated KCNE1 enhancement of *I*_f_ versus anti‐automatic influences, introduced by KCNE1‐WT under iron‐TMPRSS6‐dependent regulation. In this framework, arrhythmogenesis does not result from a simple additive effect of multiple risk variants, but from context‐dependent dominance of either depolarizing or suppressive mechanisms. Perturbations of this balance—such as increased surface TMPRSS6 expression induced via increased iron levels (Figure 5), changes in protease activity at the plasma membrane as described in a preprint [33], or disease‐related remodeling—may shift the system toward either excessive ectopic activity or impaired pacemaker function, clinically manifesting as AF, SSS, or tachycardia‐bradycardia syndrome.

How does TMPRSS6 cleave KCNE1‐WT and its variant KCNE1‐G38S? We reported that TMPRSS6 cleavage occurs in a ^32^RRSPRSS^38^ segment of KCNE1 [33]. Here, we show that KCNE1‐WT and KCNE1‐G38S are differently sensitive to TMPRSS6 cleavage in a cell‐based FACS assay (Figure 4). Positioning of target peptides relative to the proteolytic site of a protease is critical for cleavage rate. In order to understand if the positioning of KCNE1‐peptide ^32^RRSPRSS^38^ relative to the plasma membrane is critical in TMPRSS6 cleavage, the KCNE1‐^32^RRSPRSS^38^ distance relative to the outer leaflet was increased, and the effects were tested in a FACS assay. Consistently, increasing the distance of KCNE1‐^32^RRSPRSS^38^ relative to the outer plasma membrane by successive introduction of 1, 2, 3, or 4 alanines into KCNE1 between the outer membrane edge and KCNE1‐^32^RRSPRSS^38^ clearly shows that positioning of the target peptide is highly critical to TMPRSS6 cleavage in cells (Figure 5). In silico, these differences in KCNE1‐WT versus KCNE1‐G38S TMPRSS6 cleavage are conclusively mimicked by relatively extensive different binding energies of KCNE1‐WT versus KCNE1‐G38S in membranes simulating the event in a cell environment (Figure 6). However, membrane‐free simulations on reduced proteins in solution suggest that the in silico affinity of KCNE1^32–38^ regions to the TMPRSS6 PD depends on residue KCNE1^38^ to some extent. However, these differences are largely boosted in a membrane environment, suggesting that both interaction with TMPRSS6 and especially positioning of peptide KCNE1^32–38^ in relation to TMPRSS6 are impacted by residue KCNE1^38^. The increased in silico affinity of KCNE1‐WT versus KCNE1‐G38S suggests that the KCNE1‐G38S‐TMPRSS6 may be stabilized and thus the interconversion rate between bound and KCNE1‐G38S‐cleaved/released states is reduced compared to cleavage events in KCNE1‐WT.

Our findings suggest important implications for SSS, AF, and disease progression. Clinically, this model of HCN4‐linked arrhythmogenesis, based on dynamic balance rather than unidirectional gain‐of‐function in HCN4 via KCNE1, aligns well with the heterogeneous phenotypes observed in carriers of HCN4 and KCNE1 variants, ranging from sinus bradycardia to AF and TIC [16, 17, 18, 19]. Iron and TMPRSS6‐dependent reduction of HCN4 may contribute to sinus node dysfunction and bradyarrhythmic components, while residual or episodically unrestrained HCN4 activity may still permit atrial ectopy and AF initiation. Thus, alternating dominance of pro‐ and anti‐automatic mechanisms could underlie the characteristic tachycardia‐bradycardia phenotype seen in affected patients. TMPRSS6 expressed in cardiac cells could act on the channel complex, or alternatively, TMPRSS6 may become active on HCN4/KCNE1 when released from the liver and transported via the bloodstream to the heart. Similarly, other closely related serin‐arginin proteases, like TMPRSS2 (matriptase 1), show a similar target pattern and may process KCNE1 like TMPRSS6, raising the possibility that such proteases target HCN4/KCNE1 channels similarly. More research is required to elucidate this possibility.

However, our study has limitations. The mechanistic insights are derived from heterologous expression systems, which potentially cannot fully recapitulate the complexity of the human SAN and atrial cardiomyocytes. Furthermore, the regulation, including serum iron levels of TMPRSS6 activity in the human heart—both spatially and temporally—remains incompletely understood. Future studies using patient‐specific induced pluripotent stem cell‐derived cardiomyocytes and in vivo models will be essential to validate the proposed regulatory framework.

In conclusion, this study identifies TMPRSS6‐dependent suppression of HCN4 in the context of common KCNE1 variants as a novel regulatory mechanism within the cardiac pacemaker network. Our data demonstrate that disease‐associated ion channel variants do not act in isolation but are embedded in multilayered genetic and posttranslational control systems. By revealing a protease‐dependent inhibitory component that counterbalances KCNE1‐mediated HCN4 gain‐of‐function in a HCN4 disease mutant, this work provides a refined and (patho‐)physiologically plausible model of SAN dysfunction and atrial arrhythmogenesis, with important implications for individualized risk stratification and therapeutic targeting.

## 4 Experimental

### 4.1 Molecular Biology

Molecular biology methods were performed essentially as described previously. Human HCN4 cDNA (AJ132429.1) and KCNE1 (NP_000210.2) were subcloned into the *X. laevis* oocyte expression vector pSGEM or mammalian expression vector pSGEM (as described in Möller et al. [19] and Peischard et al. 2022 [23]), and the HCN4 clones were mutated at position P883R and KCNE1 at position G38S by site‐directed mutagenesis using QuikChange Lightning Site‐Directed Mutagenesis Kit (Agilent Technologies). All constructs were confirmed by automated DNA sequencing. All oocyte expression constructs for oocyte expression were linearized with NheI and used as a template for in vitro transcription of poly(A)‐tailed cRNA with the T7 mMESSAGE mMACHINE kit (Ambion). Concentrations of cRNA were examined by photospectrometry (NanoDrop ND‐100), and the quality of the transcript was verified by agarose gel electrophoresis.

### 4.2 Flow Cytometry (FACS) Analysis

HEK293T cells were cultured and transfected as previously described in a preprint [33]. For further FACS sample preparation, cells were washed with PBS, mechanically dissociated, and centrifuged at 200 × *g* for 2 min. The resulting cell pellet was washed again with PBS, resuspended in 500 µL PBS, and immediately subjected to flow cytometric analysis on a FACS Aria III flow cytometer (BD Biosciences, Franklin Lakes, NJ, USA). For each sample, 1 × 10^5^ cells were analyzed. Data acquisition and gating were carried out using FACS Diva software, version 6.1.3 (BD Biosciences) and FlowJo version 10.5.3 (BD Life Sciences). EGFP fluorescence was excited at 488 nm, and an emission wavelength filter of 530/30 nm was used. An excitation wavelength of 561 nm and an emission wavelength filter of 610/20 nm were applied for the determination of pHmScarlet [36]. The same gating strategy was applied uniformly to all samples. FSC‐A/SSC‐A gating selected intact cells, followed by FSC‐A/FSC‐H to isolate singlets. Fluorescence signals were evaluated relative to unstained cells from the same differentiation batch, which served as negative controls for background signals. Cells were gated based on green fluorescence in order to include only positive transfected cells. Red fluorescence was then analyzed directly, and the GFP‐positive cells were exported for further analysis. The exported data were assessed using R (version 4.4.2; R Core Team) and the flowCore package (version 2.18.0), where the pHmScarlet/EGFP intensity ratio (RFP/GFP ratio) per cell was calculated and averaged per transfection approach.

### 4.3 Two‐Electrode Voltage‐Clamp Recordings in *X. laevis* Oocytes

cRNA synthesis, oocyte handling, and two‐electrode voltage‐clamp (TEVC) recordings were carried out using established protocols with minor modifications [23, 39]. Briefly, plasmid cDNAs were linearized and transcribed into capped cRNA using the mMESSAGE mMACHINE T7 in vitro transcription kit (Invitrogen, Carlsbad, CA, USA). Defolliculated *X. laevis* oocytes (EcoCyte Bioscience, Dortmund, Germany) were microinjected with defined combinations of cRNA encoding HCN4 (5 ng), KCNQ1 (2.5 ng), KCNE1 (co‐expression with HCN4: 2.5 ng; with Kv7.1: 0.25 ng), and TMPRSS6 (5 ng). Following injection, oocytes were maintained for 48–72 h at 18°C in Barth's solution containing (in mM): 88 NaCl, 1 KCl, 0.4 CaCl_2_, 0.33 Ca(NO_3_)_2_, 0.6 MgSO_4_, 5 Tris‐HCl, and 2.4 NaHCO_3_, supplemented with theophylline (80 mg/L), benzylpenicillin (63 mg/L), streptomycin (40 mg/L), and gentamicin (100 mg/L). TEVC recordings were performed using microelectrodes filled with 3 M KCl (resistance 0.5–1.5 MΩ) in ND96 recording solution containing (in mM): 96 NaCl, 4 KCl, 1.8 MgCl_2_, 0.1 CaCl_2_, and 5 HEPES, adjusted to pH 7.4 with NaOH. Oocytes expressing KCNQ1 were clamped at a holding potential of −80 mV, whereas HCN4‐expressing oocytes were held at −40 mV. Two hours prior to electrophysiological recordings of the Kv7.1 channel, oocytes were incubated in Barth's solution containing the purified TMPRSS6 PD at a final activity of 170 U/mL (Human Recombinant Matriptase 2 (TMPRSS6) (NM_153609) Protein, SKU TP710211, residues 77–811 aa, with C‐terminal DDK tag, Origene).

For HCN4 currents, a voltage protocol was applied to assess voltage‐dependent activation. All electrophysiological data were acquired using the custom GePulse software (http://users.ge.ibf.cnr.it/pusch/programs-mik.htm; Dr. Michael Pusch, Genoa, Italy). For data analyses, custom ANA software (http://users.ge.ibf.cnr.it/pusch/programs-mik.htm; Dr. Michael Pusch, Genoa, Italy) or a custom code written in R Statistical Software (version 4.4.2, R Core Team) was used. For each voltage step, mean current amplitudes were determined from the last 40 ms of the respective pulse, exported as CSV files, and further statistically analyzed.

### 4.4 Molecular Modeling and MD Simulations

Molecular modeling and simulation were carried out with YASARA Structure (version 25.9.17), and quantitative analyses were performed using OriginPro 2019. The TMPRSS6‐KCNE1 complex has been modeled before in a preprint [33] and was used as described. In brief, the NMR structure of KCNE1 in lipid micelles (PDB ID: 2K21) served as the basis for the KCNE1 model. The TMPRSS6 structure was obtained by homology modeling, using the AlphaFold prediction of TMPRSS6 together with the crystal structure of matriptase‐1 bound to a peptidomimetic benzothiazole inhibitor (PDB ID: 6N4T) as templates. In the 6N4T complex, the benzothiazole ligand marks the substrate‐binding region characteristic of arginine‐specific serine proteases and pinpoints the key arginine residue that anchors the substrate side chain. The corresponding position in KCNE1 was assigned to R36, located immediately before the predicted scissile bond at S37. Consequently, the KCNE1 segment ^32^RRSPRSG/S^38^ (KCNE1 position 38 G/S represents the residues glycine or serine) was placed into the TMPRSS6 active‐site cleft, oriented according to the benzothiazole ligand in 6N4T, and the system was energy equilibrated as a membrane system by 30‐ns MD simulations. The simulations in this study used the following conditions: Membrane‐embedded MD simulations were performed with the YASARA macro *md_runmembranefast.mcr*. The protein complex was inserted into a phosphatidylethanolamine (PE) bilayer, with 1‐palmitoyl and 2‐oleoyl chain lipid headgroups. Placement of the system within the membrane employed YASARA's automatic positioning routine. All MD simulations were run with the AMBER14 force field, using timesteps of 2 × 2.5 fs and periodic boundary conditions in a cubic simulation box. Long‐range electrostatics were treated with the particle‐mesh Ewald scheme and an 8 Å cutoff. The system was solvated with *TIP3P* water at a density of 0.997 g/cm^3^, supplemented with 0.9% NaCl at pH 7.4. Simulations were conducted in the NPT ensemble at 298 K and 1 atm. At position 38 of KCNE1, glycine or serine substitutions were introduced to generate the respective variants. Each production run was continued for 331.5 ns. Structural stability was monitored via the root mean square deviation (RMSD) of the full TMPRSS6–KCNE1 complex as well as of the KCNE1^32–38^ segment, sampled every 0.25 ns during both equilibration and production. Interaction analyses and binding energy calculations were performed on snapshots taken from the production trajectories. For this purpose, the YASARA macros *md_analyze.mcr* were modified to define KCNE1^32–38^ as the ligand. Binding energies were calculated using YASARA macros *md_bindenergy.mcr* defining TMPRSS6/TMPRSS6 catalytic domain and KCNE1 variant/KCNE1 peptide as binding partners.

### 4.5 Statistical Analysis

Statistical analysis was performed using SPSS (version 31.0, SPSS Inc., Chicago, IL, USA). Normality of the pooled residuals was assessed using the Shapiro–Wilk test and the Levene test for homogeneity of variances across experimental groups. For the first TEVC experiment (Figure 1) and for the comparison of the HCN4‐TMPRSS6 effect (Figure 2E), a Welch ANOVA followed by Games–Howell post hoc comparisons was employed. The second TEVC analysis (Figure 2) was a 2 × 2 × 2 factorial design with three independent factors: Ion channel (HCN4‐WT vs. HCN4‐P883R), subunit (KCNE1‐WT vs. KCNE1‐G38S), and TMPRSS6 (with and without)—a three‐way ANOVA with HC3 heteroscedasticity‐consistent standard errors was conducted. Significant interactions were further analyzed using estimated marginal means with Bonferroni‐adjusted pairwise comparisons. FACS data was evaluated by one‐way ANOVA followed by planned post hoc tests adjusted using Bonferroni correction. All remaining two‐group comparisons were conducted using Student's *t*‐test. Significance was judged at a *p* value of 0.05. ns stands for not significant, *p* ≤ 0.05 is indicated by *, *p* ≤ 0.01 by **, and *p* ≤ 0.001 by ***.

## Conflicts of Interest

The authors declare no conflicts of interest.

## Data Availability

The data that support the findings of this study are available from the corresponding authors upon reasonable request.
